# Effects of long-term cysteamine treatment in patients with cystinosis

**DOI:** 10.1007/s00467-017-3856-4

**Published:** 2017-12-19

**Authors:** Gema Ariceta, Vincenzo Giordano, Fernando Santos

**Affiliations:** 1 Pediatric Nephrology, Hospital Universitari Vall d’Hebron, Universitat Autónoma de Barcelona, Barcelona, Spain; 2 Servicio de Nefrología Pediátrica, Hospital Universitari Vall d’ Hebron, Pg/ Vall d’Hebron 119-129, 08035 Barcelona, Spain; 3 Orphan Europe, Puteaux, France; 4 Hospital Universitario Central de Asturias & University of Oviedo, Oviedo, Spain

**Keywords:** *CTNS* gene mutations, Cystine-depleting therapy, End-stage renal disease, Extrarenal complications, Lysosomal cystine accumulation, Renal Fanconi syndrome

## Abstract

Cystinosis is a rare autosomal-recessive lysosomal storage disease with high morbidity and mortality. It is caused by mutations in the *CTNS* gene that encodes the cystine transporter, cystinosin, which leads to lysosomal cystine accumulation. Patients with infantile nephropathic cystinosis, the most common and most severe clinical form of cystinosis, commonly present with renal Fanconi syndrome by 6–12 months of age, and without specific treatment, almost all will develop end-stage renal disease (ESRD) by 10–12 years of age. Early corneal cystine crystal deposition is a hallmark of the disease. Cystinosis also presents with gastrointestinal symptoms (e.g., vomiting, decreased appetite, and feeding difficulties) and severe growth retardation and may affect several other organs over time, including the eye, thyroid gland, gonads, pancreas, muscles, bone marrow, liver, nervous system, lungs, and bones. Cystine-depleting therapy with cysteamine orally is the only specific targeted therapy available for managing cystinosis and needs to be combined with cysteamine eye drops for corneal disease involvement. In patients with early treatment initiation and good compliance to therapy, long-term cysteamine treatment delays progression to ESRD, significantly improves growth, decreases the frequency and severity of extrarenal complications, and is associated with extended life expectancy. Therefore, early diagnosis of cystinosis and adequate life-long treatment with cysteamine are essential for preventing end-organ damage and improving the overall prognosis in these patients.

## Introduction

Cystinosis is a rare autosomal-recessive lysosomal storage disease occurring approximately once every 100,000–200,000 live births, that is associated with high morbidity and mortality [1, 2]. The disease is caused by mutations in the *CTNS* gene that encodes the lysosomal cystine transporter, cystinosin, which result in the accumulation of cystine within the lysosome [3, 4]. There are three clinical forms of cystinosis: infantile or early-onset nephropathic cystinosis (OMIM 219800), juvenile or late-onset nephropathic cystinosis (OMIM 219900), and adult or ocular cystinosis (OMIM 219750) [1, 2]. Infantile nephropathic cystinosis is the most severe and most common form (~95% of cases) and that in which apparently healthy neonates usually present with renal Fanconi syndrome within the first year of life. In the absence of appropriate treatment, these patients develop end-stage renal disease (ESRD) by 10–12 years of age [5, 6]. The availability of renal replacement therapy (RRT) (i.e., dialysis and renal transplantation) since the early 1980s has improved the prognosis of patients with nephropathic cystinosis [7], but longer patient survival has highlighted the emergence of late manifestations of the disease. These extrarenal complications may develop in several organs, including the eye, thyroid gland, pancreas, gonads, lungs, muscles, brain, and bones [8–13]. Early diagnosis and adequate treatment is essential to prevent or attenuate end-organ damage and improve overall prognosis [14].

Life-long cystine-depleting therapy with cysteamine orally is the mainstay of treatment for cystinosis, and if initiated early, it can dramatically improve renal survival, patient’s quality of life, and overall disease prognosis [1, 15]. The most commonly used formulation is immediate-release cysteamine bitartrate (Cystagon®, Mylan Pharma, USA, and Orphan Europe, France), which has been on the market since the 1990s, and delayed-release cysteamine bitartrate (Procysbi®, Horizon Pharma, USA) was recently approved. Early and continuous long-term treatment with cysteamine is associated with delayed ESRD onset, a decreased risk of extrarenal complications, and improved survival rates [16, 17]. Frequency and severity of complications are decreased in patients with good compliance to cysteamine therapy [18, 19]. However, adolescents and adults tend to show lower treatment adherence after transition from pediatric to adult medical care. Given that the life expectancy of patients with cystinosis has now been increased into adulthood, as yet uncharacterized complications may emerge in well-treated adult patients with cystinosis [17].

Here we provide a brief overview of the pathogenesis of cystinosis and describe the long-term benefits of sustained cysteamine therapy, including reduced renal and extrarenal complications and increased life expectancy in patients with cystinosis.

## Pathogenesis of cystinosis

Cystinosis is caused by bi-allelic mutations in the *CTNS* gene located on chromosome 17p13.2 that encodes for cystinosin, a ubiquitously expressed membrane protein that mediates the efflux of lysosomal cystine [3, 4, 20]. The most common *CTNS* mutation in Western populations is a large ~57-kb deletion that includes the *D17S829* locus [3, 4], as well as the *CARKL* gene that encodes the enzyme sedoheptulokinase [21]. Nephropathic cystinosis is usually associated with two severe or truncating *CTNS* mutations that affect the promoter, leader sequence, transmembrane or non-transmembrane regions, including small deletions/insertions, missense and splicing mutations [1].

Although the exact mechanism of cystinosis is not fully understood, the lack of functional cystinosin is known to cause accumulation and crystallization of cystine within the lysosome, which ultimately results in apoptosis and tissue damage in all organ systems [22, 23]. Lysosomal cystine crystals are thought to cause elevated intracellular oxidative stress [24] and may activate inflammasome-related gene expression in proximal tubular epithelial cells [25]. In addition to cystine accumulation, studies have indicated that changes in the expression of transcription factors [26] and the mammalian target of rapamycin signaling pathway [27] may also play a role in the pathogenesis of cystinosis. Other mechanisms, such as the expression of abnormal cystinosin protein itself, increased apoptosis, proximal tubular cell metaplasia, and damage to podocytes, are also likely to play a role in the associated renal impairment[23, 28].

## Cysteamine therapy

**Fig. 1:**
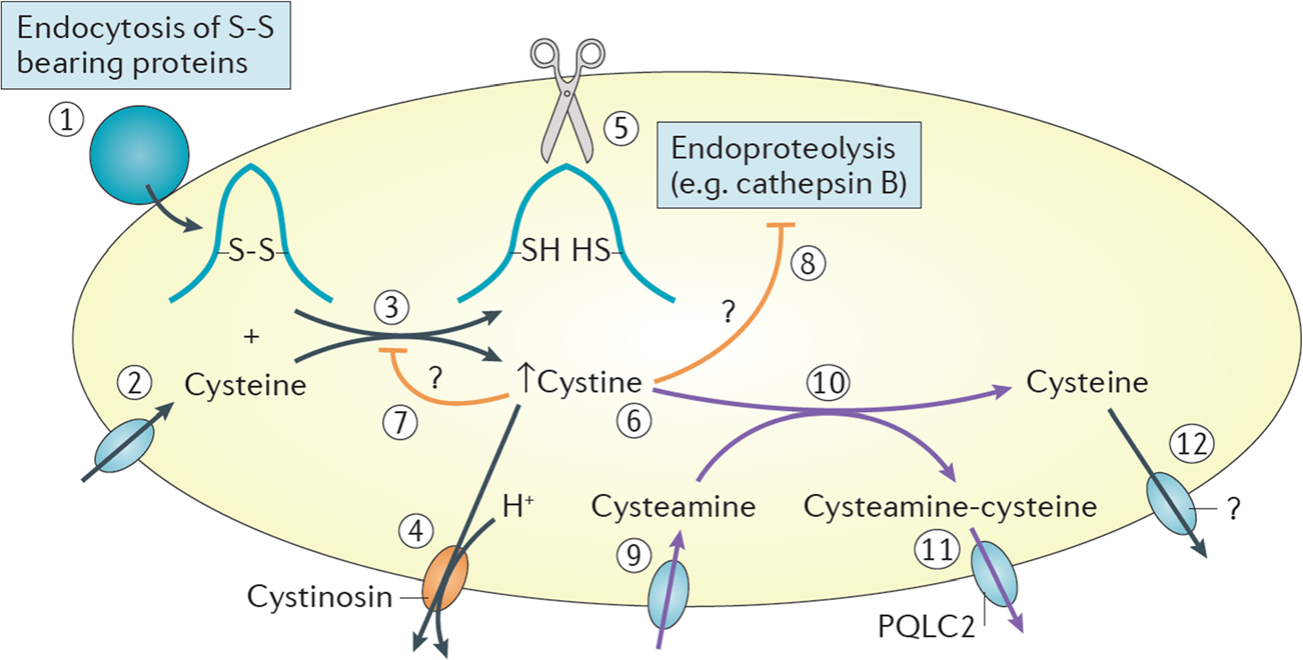
Cysteamine mechanism of action. Reproduced from Cherqui and Courtoy 2017 [23] (with permission)

Cysteamine enters the lysosome by an unknown transporter and breaks down cystine into cysteine and cysteine–cysteamine disulfide, which are removed by the cysteine transporter and the lysine/arginine (PQLC2) transporter, respectively (Fig. 1) [29, 30]. Cysteamine rapidly deplete cells and tissues of lysosomal cystine [31] and is the only specific targeted therapy available for patients with cystinosis [1]. Based on the efficacy of orally administered cysteamine in slowing renal function decline [6, 32], the drug was approved for clinical use in the 1990s.

In vitro, cysteamine is associated with restoration of glutathione redox status in cultured cystinotic renal tubular epithelial cells [33] and differentiation of mesenchymal stromal bone marrow cells [34]. Cysteamine also increases spermatogenesis [35], reduces oxidative stress [36, 37], and prevents inhibition of thiol-containing enzymes [38–40].

Cysteamine dosage calculations are based on body surface area (BSA) in adults, with a maximum dose of 1.95 g/m^2^ per day [17]. Intracellular leukocyte cystine (ILC) levels, which are typically >2 nmol half-cystine/mg protein in patients with cystinosis [1], may be monitored to optimize cysteamine dose and help identify patients with poor compliance. Target ILC trough levels of <1 nmol half-cystine/mg protein are used for adequate disease control and cysteamine dose adjustment [6]. However, it should be noted that ILC levels can vary between laboratories, and the exact benefits of measuring levels is not well established.

At higher doses (>1.95 g/m^2^ per day) of cysteamine, rare adverse effects have been reported, including bone pain, myalgia, skin striae, and bruise-like lesions on the elbows (reactive angioendotheliomatosis on skin biopsy) [41]. Cysteamine increases human dermal microvascular endothelial cell survival in vitro, which may explain how the drug causes angioendotheliomatosis [42]. As it is metabolized into volatile sulfur compounds (i.e., dimethyl sulfide and methanethiol), treatment is also associated with an unpleasant sulfurous body and breath odor, which can often lead to poor treatment compliance [41].

Other cysteamine-related adverse events include allergic rash, hyperthermia, lethargy, neutropenia, seizures, and gastrointestinal discomfort; however, these effects are reversible, and most are prevented by starting the drug at a low dosage with gradual titration as tolerated under the guidance of experienced physicians [43]. As cysteamine toxicity may be associated with copper deficiency in patients with renal Fanconi syndrome, copper supplementation may prevent cysteamine-related adverse effects in some patients [44].

## Long-term effects of cysteamine therapy

Long-term cysteamine treatment orally is associated with improvements in renal and extrarenal complications. A summary of clinical data showing the long-term effects ofshown in Table 1. Where reported [5, 45, 47], the duration of follow-up ranged from 8 to 25 years, and the largest study comprised data from 208 patients [49]. Of note, follow-up with early treatment initiation is currently limited to <30 years; therefore, the effects of cysteamine in older patients are unknown. It should also be noted that, in some studies, the lack of adequate cysteamine therapy may be due to insufficient access to treatment (e.g., in developing countries), as well as poor treatment compliance.

**Table 1:** Clinical data showing the long-term effects of cysteamine therapy orally in patients with cystinosis

First author, year	Study description	Renal outcomes	Extrarenal complications
Brodin-Sartorius, 2012 [45]	French study; adults (aged ≥15 years) diagnosed between 1961 and 1995; mean follow-up 24.6 years (*n* = 86)	• Initiation <5 years of age vs later delayed ESRD onset (mean age at onset 13.4 vs 9.6 years; *p* < 0.05) • Increased treatment compliance also delayed ESRD onset (*p* < 0.0001)	• Initiation <5 years of age (vs later or no treatment) reduced rates of hypothyroidism (52.5% vs 73.3% and 96.8%), diabetes (27.5% vs 64.7% and 89.7%), neuromuscular disorders (15% vs 53.6% and 61.1%), and death (5% vs 42.9% and 63.6%) [*p* < 0.001 for each parameter]
Broyer, 2008 [46]	Necker Enfants-Malades Hospital series; patients born before 1988; aged 20–39 years (*n* = 56)	• Initiation <3 years of age vs later delayed ESRD onset (mean age at onset 17.4 vs 9.6 years)	• Initiation <3 years of age vs later: - Improved linear growth (mean height 167 vs 144.5 cm for boys; 153.5 vs 132 cm for girls) and visual acuity (mean 8.5 vs 5) • Reduced rates of glucose intolerance, thyroxine requirements (35.7% vs 88.2%), myopathy (0% vs 38.5%), cerebellar/pyramidal symptoms or mental deterioration (0% vs 35.9%), and hepatosplenic disorders (0% vs 92.3%)
Gahl, 2007 [16]	US database study; adults (aged 18–45 years) examined between January 1985 and May 2006 (*n* = 100)	• Treatment for ≥8 years vs <8 years delayed renal transplantation (mean age 14.8 vs 11.0 years)	• Increased duration of therapy (from <10 to >20 years) reduced rates of diabetes (from 28 to 0%), myopathy (from 60 to 0%), pulmonary dysfunction (from 80 to 0%), and death (from 43 to 0%) • Treatment for ≥8 vs <8 years significantly improved mean height (154.7 vs 143.6 cm) and weight (53.2 vs 45.3 kg) parameters, and rates of hypothyroidism (56% vs 87%) and death (8% vs 49%)
Greco, 2010 [5]	Italian single-center study; patients diagnosed at 3–60 years of age; median follow-up 17.6 years (*n* = 23)	• Initiation <2.5 years of age vs later improved evolution of renal function (*p* = 0.006), and decreased risk of CKD stage 3 • Rapid renal function decline prevented by higher cysteamine doses and use of ACEi	• Patients treated more recently (initiated <2.5 years of age) had improved linear growth curves vs older children (*p* = 0.04)
Gultekingil Keser, 2014 [47]	Turkish, retrospective, single-center study; patients aged 0.5–29 years; median follow-up 8 years (*n* = 21)	NR	• Patients with ILC levels <2 vs ≥2 nmol half-cystine/mg protein had reduced rates of short stature (66.6% vs 90.9%), pubertal delay (0% vs 66.6%), hypothyroidism (33.3% vs 54.5%), and diabetes (0% vs 18.1%)
Nesterova, 2015 [19]	US database study; patients aged 11–48 years examined between 1975 and 2005 (*n* = 147)	• Treatment compliance was linearly correlated with age at ESRD onset (*R*^2^ = 0.997) • Optimal treatment for 1 year preserved 0.9 years of renal glomerular function • Severity of renal Fanconi syndrome (decreased tubular function) was not affected	NR
Sonies, 2005 [48]	US database study; patients aged 6–45 years examined between February 1987 and March 2004 (*n* = 101)	NR	• Severity of swallowing dysfunction (muscle atrophy) decreased with increased number of years of treatment
Tsilou, 2006 [49]	US database study; patients aged 0.5–42 years examined between 1976 and 2004 (*n* = 208)	NR	• Treatment for >20 vs ≤10 years decreased rate of retinopathy (0% vs 28%)
Vaisbich, 2010 [50]	Brazilian Multicenter Nephropathic Study; patients aged 1.3–29 years enrolled since 1999 (*n* = 102)	• Initiation <2 years of age vs later reduced rate of CKD stage II–V (25% vs 77.5%)	• Initiation <2 years vs later: • Improved growth (weight and height) parameters • Reduced rates of hypothyroidism (10% vs 61.7%), diabetes (5% vs 7.8%), muscular weakness (0% vs 6.8%), hepatic dysfunction (0% vs 4.9%), CNS disorders (0% vs 4.9%), and swallowing dysfunction (0% vs 1.9%)
Viltz, 2013 [51]	Children and adolescents (aged 3–18 years) [*n* = 46]	NR	• Initiation <2 years vs later improved cognitive function (verbal, performance, and full-scale IQ scores, and spatial-relations test; *p* < 0.05), but not visual–motor performance scores

*ACEi* angiotensin-converting enzyme inhibitor, *CKD* chronic kidney disease, *CNS* central nervous system, *ESRD* end-stage renal disease, *ILC* intracellular leukocyte cystine, *IQ* intelligence quotient, *NR* not reported

## Renal outcomes

Although cysteamine does not significantly affect renal Fanconi syndrome in most patients [1, 2], in patients with early initiation and good treatment compliance, long-term cysteamine therapy improves the evolution of renal function in patients with cystinosis, delaying progression of glomerular function deterioration, ESRD onset, and the need for renal transplantation [5, 16, 19, 45, 46, 50]. Early initiation was associated with preserved renal function and reduced incidence of chronic kidney disease in both a Brazilian multicenter study [50] and a long-term retrospective study [5]. In the latter study, higher doses of cysteamine and the use of angiotensin-converting enzyme inhibitors (ACEis) were also significantly associated with a delay in the decline of renal function [5]. Early initiation and adequate long-term cysteamine therapy delayed the onset of ESRD in the Necker-Enfants Malades Hospital series of patients with cystinosis, who survived to at least 20 years of age [46], and in a French study of adults [45]. Increased compliance to cysteamine therapy also had a linear correlation with the mean age at ESRD onset (*R*^2^ = 0.997) in a long-term retrospective study [19].

Taken together, these studies confirm that cysteamine therapy orally must be initiated as early as possible following diagnosis, even in small infants, and be used long term with good compliance to maximize its beneficial effects on delaying progression of renal failure and ESRD onset.

## Extrarenal complications

In long-term studies, patients who initiated treatment early [5, 45, 46, 50, 51] and received adequate cysteamine therapy [16] had increased life expectancy and fewer complications than inadequately treated patients and those who started treatment later or were untreated (Table 1). Given the strong evidence for cysteamine-associated benefits and longer life expectancy in most patients, there is increasing interest in improving patient care with regard to extrarenal disease manifestations, treatment adherence, and adult patient care.

### Eye disorders

Untreated patients with infantile nephropathic cystinosis develop corneal cystine crystal accumulation that leads to ocular discomfort and photophobia [1, 13]. As cysteamine therapy orally has no effect on corneal cystine crystals, cysteamine eye drops are used to dissolve crystals and reduce symptoms. A new topical viscous formulation of cysteamine eye drops has been developed that requires less frequent instillation and is more effective than standard cysteamine eye drops [52].

Intracellular cystine crystal deposition can also occur in posterior-segment ocular structures and is associated with damage to retina, choroid, and optic nerve [13, 49]. Long-term cysteamine therapy orally may reduce posterior-segment complications. In a retrospective US National Institutes of Health (NIH) study, a decreased incidence of retinopathy from 28 to 0% was observed among patients with cystinosis when therapy was increased from ≤10 years to 21–30 years [49].

### Endocrine disorders

Long-term cysteamine therapy orally is associated with marked improvements in growth retardation and other endocrine disorders [5, 16, 46, 47, 50]. Growth hormone therapy may also improve growth outcomes [1]. Early initiation and long-term therapy led to significantly improved mean height [16, 46] and weight [16, 50] parameters in several studies. Early initiation was also associated with improved linear growth curves in an Italian study, where poorer linear growth was observed in older children (in whom cysteamine was started after 2.5 years of age and suboptimal treatment was more likely) compared with those who initiated cysteamine more recently [5]. Early therapy initiation is also associated with a lower incidence of hypothyroidism and diabetes mellitus [46, 50] and significant delays in the onset of these conditions [45]. Furthermore, long-term (≥8 years) cysteamine therapy reduces the incidence of hypothyroidism and diabetes in adults with cystinosis [16].

A long-term study (median follow-up 8 years) indicated that good compliance to cysteamine therapy orally is effective in preventing endocrine disorders in patients with cystinosis, with ILC levels of <2 nmol half-cystine/mg protein being associated with lower incidences of short stature, pubertal delay, hypothyroidism, and diabetes than those with higher ILC levels [47].

### Neurocognitive disorders

Cystinosis is associated with several neurologic complications, including structural brain abnormalities, cognitive impairment, and learning difficulties [8]. Long-term studies have indicated that early initiation of cysteamine orally may prevent many of these neurocognitive disorders [46, 50, 51]. Cerebellar or pyramidal symptoms or mental deterioration [46] or central nervous system disorders [50] were only observed among patients who started cysteamine after 2 [50] or 3 [46] years of age. Early initiation (<2 years of age) is also associated with significantly higher performance, verbal, and full-scale IQ scores, and significantly improved visual–spatial skills, although visual–motor skills were not significantly affected [51].

### Muscular disorders

Distal myopathy and muscle atrophy are significant complications of cystinosis among adolescents and young adults, particularly in untreated patients and those with poor compliance to cysteamine therapy [9]. The frequency and severity of these complications can be reduced with long-term cysteamine, if initiated early. In adults, long-term (>20 years) therapy was associated with a decrease in the incidence of myopathy (to 0%) compared with a treatment duration of ≤10 years (60%) [16], and the incidence of neuromuscular complications decreased significantly among patients who initiated therapy early (<5 years) [45]. In two studies, oropharyngeal dysfunction and/or distal muscle atrophy were only found in patients who started therapy >2 [50] or 3 [46] years of age. Swallowing dysfunction, which occurs due to muscle atrophy and increases the risk of fatal aspiration, decreased in severity with increased duration of treatment [48].

### Pulmonary disorders

Patients with cystinosis may develop pulmonary complications, including respiratory failure caused by muscle weakness, sleep apnea, and aspiration due to swallowing dysfunction [10]. In adults, longer-term cysteamine therapy is associated with a lower incidence of pulmonary disorders [16]. Mean forced vital capacity was higher among patients who received cysteamine for >10 years (83% of predicted) than in those with ≤10 years of therapy (54% of predicted) [16].

## Mortality

Long-term cysteamine therapy is associated with improved survival rates among patients with cystinosis, particularly when initiated early [16, 45, 46]. In adults, long-term cysteamine was associated with lower mortality rates, with the incidence of death decreasing as the duration of therapy increased [16]; in other studies, lower rates of mortality were observed among patients who started therapy early (<3 [46] or 5 [45] years).

## Treatment adherence

The benefits of long-term cysteamine therapy are most evident in patients with good compliance to treatment and are related to the duration of therapy [18, 19]. In a retrospective NIH study, each year of excellent cystine-depleting therapy resulted in almost 1 year of preserved renal function [19]. Treatment tends to be most successful in children aged <11 years, as they have the highest treatment adherence, largely due to administration of cysteamine being conducted by a parent [18].

## Conclusions

Inadequately treated cystinosis can lead to multisystem morbidity and mortality. However, long-term cysteamine therapy, if initiated early and used with good treatment compliance, provides significant improvements in renal and extrarenal outcomes. Early diagnosis and adequate long-term treatment are therefore essential for preventing end-organ damage and improving the overall prognosis in these patients. Major efforts to maintain and promote adequate long-term cysteamine therapy should be undertaken by healthcare professionals, patients, and supportive groups.
